# WHO PLANS FOR LATER LIFE? AN EXAMINATION OF MIDDLE-AGED AND OLDER FLORIDIANS

**DOI:** 10.1093/geroni/igac059.2012

**Published:** 2022-12-20

**Authors:** Anne Barrett, Hope Mimbs, Cherish Michael, Jessica Noblitt

**Affiliations:** Florida State University, Tallahassee, Florida, United States; Florida State University, Tallahassee, Florida, United States; Florida State University, Tallahassee, Florida, United States; Florida State University, Tallahassee, Florida, United States

## Abstract

The extension of life expectancy highlights the importance of understanding how people conceptualize – and plan for – their later years. We address this issue using data from an online survey of over 3,400 Floridians aged 50 and older that was conducted between December 2020 and March 2021 and funded by the Florida Department of Transportation. We examine five types of planning: for health care needs, financial well-being, living arrangements, driving retirement, and end-of-life care. We find that the likelihood of planning varies considerably across these types. Only 23 percent of respondents reported planning “some” or “a lot” for driving retirement, compared with 74 percent for health care needs, 76 percent for end-of-life care, 77 percent for living arrangements, and 83 percent for financial well-being. Likelihood of planning varied by age, gender, socioeconomic status, health, and race or ethnicity. Across all types of planning, older adults and those with at least a college degree and higher income were more likely to have planned. Women were more likely than men to plan for their financial future, living arrangements, driving retirement, and end-of-life care. Those in better health were more likely to plan for their financial future and end-of-life care. The effects of race or ethnicity were less consistent across the types of planning. White respondents were more likely than other race or ethnic groups to report planning for their living arrangements and end-of-life care, while Hispanic respondents were more likely than other groups to plan for driving retirement.

